# The Embryonic Key Pluripotent Factor NANOG Mediates Glioblastoma Cell Migration via the SDF1/CXCR4 Pathway

**DOI:** 10.3390/ijms221910620

**Published:** 2021-09-30

**Authors:** Ana Virginia Sánchez-Sánchez, Antonio García-España, Pilar Sánchez-Gómez, Jaime Font-de-Mora, Marián Merino, José Luis Mullor

**Affiliations:** 1 Bionos Biotech, SL, Biopolo Hospital La Fe, Av. Fernando Abril Martorell 106, 46026 Valencia, Spain; sanchezav78@gmail.com (A.V.S.-S.); mmerino@bionos.es (M.M.); 2 Research Unit, Hospital Universitari de Tarragona Joan XXIII, Institut d’Investigació Sanitària Pere Virgili, Universitat Rovira i Virgili, 43005 Tarragona, Spain; antoniogem85@gmail.com; 3 Neurooncology Unit, Instituto de Salud Carlos III-UFIEC, Crtra/Majadahonda-Pozuelo, Km 2, Majadahonda, 28220 Madrid, Spain; psanchezg@isciii.es; 4 Laboratory of Cellular and Molecular Biology, Instituto de Investigación Sanitaria Hospital La Fe, 46026 Valencia, Spain; jaime.fontdemora@gmail.com

**Keywords:** NANOG, CXCR4, cancer stem cell, cancer cell migration, glioblastoma

## Abstract

NANOG is a key transcription factor required for maintaining pluripotency of embryonic stem cells. Elevated NANOG expression levels have been reported in many types of human cancers, including lung, oral, prostate, stomach, breast, and brain. Several studies reported the correlation between NANOG expression and tumor metastasis, revealing itself as a powerful biomarker of poor prognosis. However, how NANOG regulates tumor progression is still not known. We previously showed in medaka fish that Nanog regulates primordial germ cell migration through Cxcr4b, a chemokine receptor known for its ability to promote migration and metastasis in human cancers. Therefore, we investigated the role of human NANOG in CXCR4-mediated cancer cell migration. Of note, we found that NANOG regulatory elements in the *CXCR4* promoter are functionally conserved in medaka fish and humans, suggesting an evolutionary conserved regulatory axis. Moreover, CXCR4 expression requires NANOG in human glioblastoma cells. In addition, transwell assays demonstrated that NANOG regulates cancer cell migration through the SDF1/CXCR4 pathway. Altogether, our results uncover NANOG-CXCR4 as a novel pathway controlling cellular migration and support Nanog as a potential therapeutic target in the treatment of Nanog-dependent tumor progression.

## 1. Introduction

The homeodomain-containing transcription factor Nanog is a crucial determinant of pluripotency [1,2,3,4]. Experiments for the reprogramming of somatic cells to ground state pluripotency revealed the key role of Nanog at different levels of this process. At the initial level, Nanog is dispensable, however, it is necessary for dedifferentiated intermediates to reach the ground state pluripotency [5]. In this regard, Nanog intervenes by overcoming reprogramming barriers and enabling self-renewal of stem cells [6,7]. In contrast to most genes associated with pluripotency, Nanog shows poor sequence identity among species, but is functionally conserved in vertebrates, suggesting that control of pluripotency resides in a unique DNA responsive element [8,9].

The hierarchical hypothesis for tumor development supports stemness features in a subpopulation of cancer cells that contribute to maintain the tumor mass from their specific niche. Cancer stem cells have been broadly studied and strong evidence supports their presence in many types of tumors [10,11,12,13,14,15,16,17]. These cancer stem cells are responsible for resistance to therapy resulting in the relapse of minimal residual disease. Moreover, Nanog expression in cancer stem cells has been described in lung, oral, prostate, stomach, breast, bladder, pancreatic, ovarian, liver and brain cancers [18,19,20,21,22,23,24,25,26,27,28,29,30]. Moreover, Nanog expression has been correlated with poor prognosis in some cancer types [29,31,32,33]. Thus, Nanog expression during cancer progression may represent a potential target for pharmaceutical intervention.

CXCR4 (CD184) is a seven-transmembrane G-protein-coupled chemokine receptor known for its ability to mediate the metastasis of a variety of cancers [34,35,36,37,38,39]. Of note, we have previously shown that Nanog regulates primordial germ cell migration through Cxcr4 in medaka fish [40], suggesting that Nanog-dependent tumor development may require CXCR4 expression. Here we show that NANOG regulatory elements in the CXCR4 promoter are functionally conserved in medaka fish and humans. In addition, transfection studies in human glioblastoma cell lines revealed the requirement of NANOG to express CXCR4. Moreover, we demonstrate that NANOG regulates *CXCR4* expression and promotes cancer cell migration through the SDF1/CXCR4 pathway. Based on the role of Nanog in cancer stem cells, our results provide a novel connection between CXCR4 and cancer stemness that may be beneficial for improving the decisions in the selected therapy.

## 2. Results

### 2.1. Medaka Fish and Mouse Display a Similar Profile of NANOG-Targeted Genes

**Figure 1:**
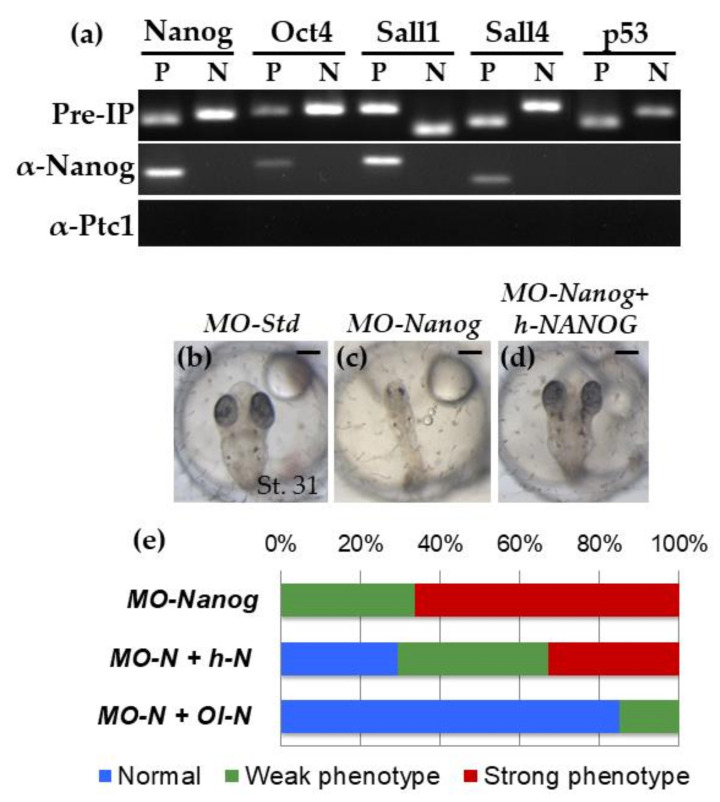
Ol-Nanog is functionally equivalent to human NANOG. (**a**) Nanog transcription factor binds to some genes at their promoters in medaka as it does in mESC. ChIP assay using the Ol-Nanog antibody. To determine the promoter sequences of the genes the conservation analysis was made using the genome sequences of Medaka, Stickleback, Tetraodon, Fugu, and Zebrafish. ChIP was performed with antibodies for Ol-Nanog, and Ol-Ptc1 as negative control. PCR reactions were performed before (pre-IP; positive control) and after ChIP. Abbreviations: Anti-Ptc1, Anti-Patched1; Pre-IP, pre-immunoprecipitation, P: regulatory region of the gene, N: sequence outside the regulatory region of the gene. (**b**–**e**) Ol-Nanog and h-NANOG rescue the abnormal phenotype provoked by MO-Nanog. (**b**–**d**) Pictures showing stage 31 embryos after injection in one cell stage of MO-Std as a MO control (**b**), MO-Nanog (**c**) and MO-Nanog+h-NANOG mRNA (**d**). While 100% of embryos injected with MO-Nanog that survive to stage 31 had small size and abnormal development of the head and trunk structures, 29.4% of embryos injected with MO-Nanog + h-NANOG showed normal size and head and trunk structures, when compared to embryos injected with MO-Std. Scale bars: 200 μm.

Murine Nanog directly regulates the expression of *Oct4, Sall1* and *Sall4*, and regulates itself in mouse embryonic stem cells (ESC) by binding to their promoter regions [41]. In contrast, Nanog does not regulate the expression of the tumor suppressor *p53*, another downstream target of this transcription factor. To study whether the gene-targeted profile by Nanog in medaka fish resembles that of mammals we performed the chromatin immunoprecipitation assay (ChIP) in medaka fish embryos with Nanog specific antibodies. Our results obtained by ChIP showed that Nanog also binds to the regulatory sequences of *Nanog, Oct4, Sall1* and *Sall4,* but not to that of *p53* (Figure 1a and Supplementary Table S1 for oligo sequences used in the experiment). Therefore, the profile of Nanog-targeted genes is similar between medaka and mice.

### 2.2. Human NANOG Rescues Nanog Knock-Down in Medaka Embryos

To study whether human *NANOG* is functionally equivalent to the predicted medaka’s ortholog we tested the ability of human NANOG to rescue Nanog knock-down phenotype in medaka embryos. We have previously described the phenotype of medaka embryos with depleted Nanog protein using a specific morpholino (MO) against Nanog’s starting codon [42]. Injection of *MO-Nanog* resulted in no normal embryos, showing strong phenotype (66.25% of the embryos with absence of embryonic body and early embryo lethality; Figure 1b,c) or weak phenotype (33.75% of embryos with small size and abnormal development of the head and trunk structures).

To perform rescue studies, we coinjected *MO-Nanog* with either *h-NANOG* or *Ol-Nanog* mRNA. Coinjection with *Ol-Nanog* mRNA resulted in moderate rescue (15% weak phenotype) or complete rescue (85% normal phenotype) of the *MO-Nanog* phenotype and embryonic viability (Figure 1e). *h-NANOG* coinjection also rescued the Ol-Nanog-depleted phenotype, where 29.4% were normal embryos (Figure 1d,e) and 37.75% were embryos with weak phenotype, but only 32.85% of embryos showed strong phenotype. Therefore, coinjection with *h-NANOG* halved the strong phenotype by rescuing the loss of Ol-Nanog function in medaka embryos. These results suggest that the predicted medaka’s Nanog is the functional ortholog gene of human *NANOG* despite the low sequence similarity.

### 2.3. Human NANOG Efficiently Binds to CXCR4 Promoter Region

**Figure 2:**
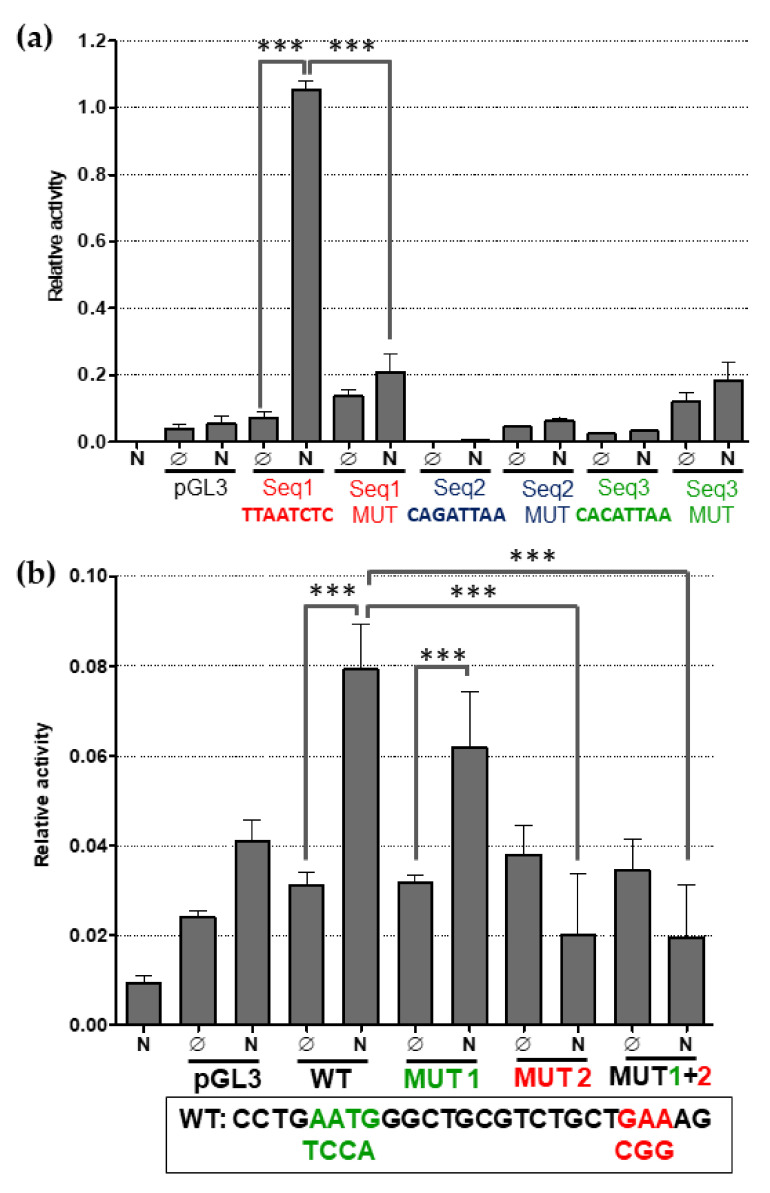
CXCR4 is a direct target of NANOG in human and medaka. Bar graphs showing reporter activities. (**a**) Regulation of luciferase reporter by Ol-Nanog. Ol-Nanog activates expression of luciferase only when it binds to the binding site sequence of Ol-Cxcr4b number 1 but this activation is not observed when assays are performed with sequences 2 and 3 or mutated sequence 1. (**b**) Regulation of luciferase reporter by h-NANOG. h-NANOG activates expression of luciferase when it binds to the binding site sequence of h-CXCR4 but this activation is not observed when the binding sequence presents the mutation 2. Abbreviations: Seq, sequence; MUT, mutation; WT: wild type sequence. *** corresponds to *p* < 0.001.

We recently reported that Ol-Nanog regulates the medaka *Cxcr4* gene expression by directly binding to its promoter [40]. Hence, we next analyzed in silico 2300 bp upstream sequence of *Ol-Cxcr4* gene using published Nanog consensus binding sequences. These analyses yielded three potential Ol-Nanog binding sequences, (sequences 1–3; (G/A) (G/C) ATTA (G/A/T) (GC); Supplementary Figure S1). Nanog binding to these sequences was further validated by dual luciferase assays. Only sequence 1 increased luciferase activity in the presence of Ol-Nanog (Figure 2a). This increment was significantly prevented by substituting two highly conserved A by C (Figure 2a) [43]. This result predicts a putative Nanog binding site in the mutated sequence of *Ol-Cxcr4* promoter (position: 781, 445–781, 452; Supplementary Figure S1).

To detect the existence of putative NANOG binding sites in the human *CXCR4* gene, we analyzed its 8530 bp upstream region. Although these searches yielded several NANOG putative binding sites, multiple searches converged on a particular 26 bp DNA stretch located within −572 and −546 from the human CXCR4 gene (Supplementary Figure S2a). To validate the functionality of this predicted h-NANOG binding site, we subcloned the sequence upstream of the luciferase gene. Luciferase assays showed a significant increase in the activity only when h-NANOG was present (Figure 2b). Since this sequence contains two putative NANOG binding motifs, we mutated each one of them individually or in combination (Supplementary Tables S1 and S2). The results revealed a major role of sequence 2 in the binding of NANOG (single or in combination with mutated sequence 1; Figure 2b). Interestingly, sequence 2 is more similar than sequence 1 to the NANOG consensus binding site reported for mouse *Rex-1 (Zfp-42)*, and human *CDK6* and *CDC25A* genes (Supplementary Figure S2b) [44,45]. Replacement of A by G in this NANOG binding site also abolished the reporter activity (Figure 2b). Thus, our results further support *CXCR4* as a direct target of h-NANOG harboring the binding site at the position chr2: 136, 876, 179–136, 876, 181.

### 2.4. h-NANOG Regulates CXCR4 Expression in Human Glioblastoma Cell Lines

**Figure 3:**
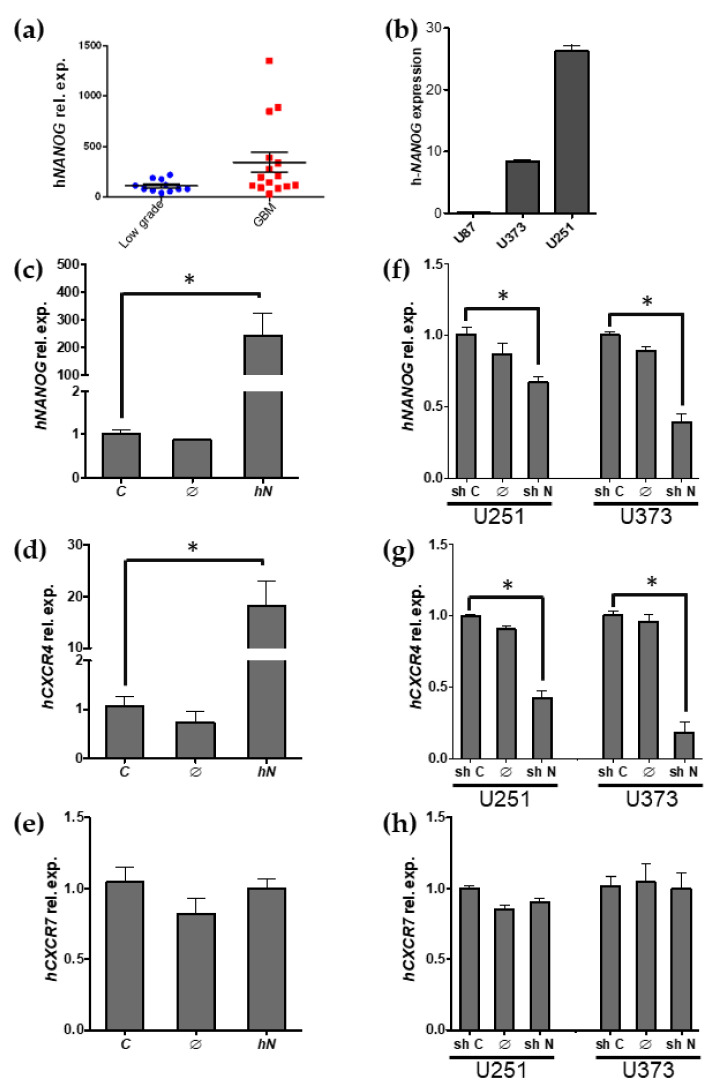
h-NANOG regulates h-CXCR4 expression but not h-CXCR7 expression in human glioblastoma-astrocytoma cell lines. Bar graphs showing relative gene expression. (**a**) h-NANOG expression in brain tumors from human patients, divided into low grade tumors or glioblastomas (GBM). (**b**) h-NANOG expression in the three glioblastoma cell lines used in this study. (**c**–**e**) Gene relative expressions of h-NANOG (**c**), h-CXCR4 (**d**) and h-CXCR7 (**e**) after h-NANOG transfection of U-87 cells. (**f**,**g**) Gene relative expressions of h-NANOG (**f**), h-CXCR4 (**g**) and h-CXCR7 (**h**) after h-NANOG transduction of U-251 and U-373 cells. Abbreviations: hN, h-NANOG; C, control; shN, shRNA-hNANOG; shC, shRNA-control. * corresponds to *p* < 0.001.

Nanog is expressed in various kinds of human tumors, including glioblastoma (Figure 3a), the most common malignant primary brain tumors [18,46,47]. On the other hand, CXCR4 controls the migration of neuronal cells [48,49], and it is the most common chemokine receptor expressed by many cancer cells, including glioblastoma [36,50], where CXCR4 plays a role in cancer cell migration [51]. To find out if h-NANOG can regulate *CXCR4* expression in human cancer cells, we studied how changes in h-NANOG level affected the expression of *CXCR4* in human glioblastoma and astrocytoma cell lines.

We used U-87, U-251 and U-373, three cell lines expressing *h-NANOG* but at different levels. U87 has a very low h-NANOG expression while the other two cell lines display a higher expression level (Figure 3b). Using q-PCR analysis we observed that ectopic expression of *h-NANOG* in U-87 cells significantly increased *NANOG* expression (Figure 3c) and correlated with increased *CXCR4* expression (Figure 3d), although the augmented expression of both genes had different ranges of increase, probably due to the post-transcriptional and post-translational regulation of *NANOG*. When we focused on *CXCR7* expression, used in this experiment as a negative control, because there is no scientific evidence of NANOG regulating the expression of this gene, results showed that CXCR7 expression did not change after *NANOG* transfection (Figure 3e).

Additionally, to analyze the effect of reduced expression of *h-Nanog* in cells that normally express higher levels of this gene, we infected U-251 and U-373 cells with Non-silencing-GIPZ lentiviral shRNAmir and GIPZ-shNanog, together with the 2nd generation packaging vectors: psPAX2 and pMD2.G. Results showed that shRNA lentiviral infection of U-251 and U-373 cells efficiently reduced *NANOG* expression levels in both cell lines (Figure 3f). Under these conditions, *CXCR4* expression levels (Figure 3g), but not of *CXCR7* (Figure 3h), were also downregulated. Taken together, the results strongly suggest that h-NANOG regulates *CXCR4* expression in human malignant gliomas.

### 2.5. h-NANOG Regulates Human Tumor Cell Migration through CXCR4

Nanog regulates migration of medaka primordial germ cells through *Ol-Cxcr4* during embryo development. In addition, *CXCR4* expression is regulated by NANOG in glioblastoma cells. Therefore, we next tested whether human NANOG could regulate migration of tumor cells through *CXCR4*.

**Figure 4:**
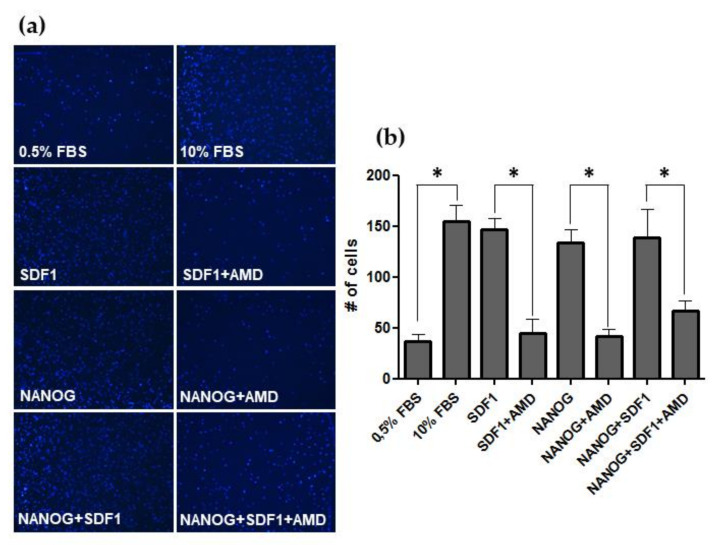
h-NANOG regulates human tumor cell migration through CXCR4. (**a**) Transwell membranes showing migrated cell nuclei stained with DAPI. (**b**) Bar graphs showing the number of cell that migrates in response of different conditions. Cells migrated in higher proportion attracted by a greater concentration of serum (10%, positive control), by the presence of SDF1, NANOG or both. The increased migration due to SDF1 or NANOG was inhibited by the presence of AMD3100 (AMD), a specific inhibitor of CXCR4 receptor. * corresponds to *p* < 0.001.

Transwell migration assays with U-87 cells showed a marked dependency on serum as a chemoattractant (Figure 4). U-87 cells were also attracted by SDF1, the chemokine of CXCR4 receptor. U-87 cells previously transfected with *h-NANOG* also showed an increase in motility (Figure 4). Notably, U-87 cell motility was suppressed in all conditions where AMD3100, a specific inhibitor of CXCR4 receptor [52], was present (Figure 4). Finally, in the presence of SDF1, no significant differences were found in cell motility between non-transfected or *h-NANOG* transfected cells, probably because of a threshold level of migration under our culture conditions as well as the starting density of the cells. Taken together, our results reveal *h-NANOG* as a mediator of cellular migration through the SDF1/CXCR4 pathway by regulating *CXCR4* expression.

## 3. Discussion

In the present study, we showed that the *CXCR4* promoter has functional responsive elements for h-NANOG. In addition, we found a correlation between *NANOG* and *CXCR4* expression levels in tumor cell lines, further supporting a role for CXCR4 in NANOG-expressing glioblastomas. Moreover, using transwell assay, we found increased cellular migration in U-87 cells transfected with *h-NANOG*. Cellular migration was abolished when a CXCR4 inhibitor was added to the cells. This result reveals that human cell migration due to NANOG is mediated by CXCR4.

The regulation of the Cxcr4 promoter by Nanog in medaka fish and humans shows high similarities in their responsive elements. In addition, human NANOG can also rescue the phenotype of medaka embryos injected with medaka-specific-morpholino. These results strongly suggest that this is a conserved transcriptional regulation in the evolution of vertebrates. Despite this conservation, the Nanog/Cxcr4 axis may be cell type dependent and trigger different cellular responses within the same species. For example, in postnatal bone development, Cxcr4 regulates osteoblast differentiation in cooperation with BMP signaling [53]. On the other hand, regarding BMP-induced mesoderm differentiation of embryonic stem cells, Nanog interacts with Smad1 interfering with the formation of active transcriptional complexes [54], therefore it seems that there could be an antagonist relation in mesodermal cells. Moreover, Cxcr4 signaling may differ between normal or pathological conditions. In fact, although Cxcr4 is required for osteoblastic differentiation, it also seems to intervene, together with YY1 and VEGF, in the pathogenesis of the malignant phenotype of osteosarcoma by promoting cell invasiveness and metastasis [55].

Recurrent cancer is associated with resistance to therapy and metastasis and is thought to be dependent on the presence of cancer stem cells, which are highly tumorigenic and resistant to chemotherapy. Several lines of evidence suggest that cancer stem cells reside in specific niches that preserve their integrity through the interaction via adhesion molecules and exchange of molecular signals [56]. In this regard, expression of the CXCR4 ligand SDF1 by specialized endothelial cells, acts as a chemoattractant for circulating malignant cells, thus intervening in early metastatic tumor spread [57]. In gliomas SDF1 is also expressed in pseudpallisading areas and microvasculature, two regions associated with cancer stem cells [58]. Moreover, in gliomas, the inhibition of CXCR4 leads to the disruption of the sonic hedgehog (SHH)-GLI-NANOG network [59], and is crucial for maintaining the self-renewal, proliferation, therapeutic resistance, and angiogenesis of glioblastoma cells in rat [60]. All these results support a role for Nanog expressing cells in colonizing specific niches. In addition, hypoxia has been shown to help maintain multiple normal stem cell populations and is also a critical microenvironmental factor in regulating self-renewal of cancer stem cells, partially by enhancing the activity of stem cell factors like Oct4, c-Myc and Nanog [61]. The stem cell niche of low oxygen upregulates the expression of *NANOG* [62] and these conditions of hypoxia are also important for the maintenance of glioblastoma stem cells [63]. NANOG is also transcribed by GLI1 activation, and they both constitute an axis that promotes stemness and growth in gliomas [46]. Of note, CXCR4 expression levels are negatively regulated under normoxic conditions by the von Hippel-Lindau tumour suppressor protein pVHL [64]. Conversely, our results show that NANOG is a transcriptional activator of CXCR4 expression, suggesting a role for NANOG in tumor progression. In addition, Nanog is a key transcriptional factor in the maintenance of normal cell stemness [6,7], and its overexpression in cancer cells is microenvironment-dependent and correlates with malignancy [14]. NANOG induction promoted drug resistance in the breast cancer cell line MCF-7, and tumor regeneration and resistance to androgens starvation in the prostate cancer cell lines Du145 and LNCaP [22]. NANOG expression has also been correlated with poor prognosis [31,32,33] and promotes breast cancer tumorigenesis and metastasis [27]. Taking into account these affirmations, it will be very interesting to assess the role of NANOG and *CXCR4* in other cancer cell lines apart from glioblastoma, like breast cancer cells. 

Our research opens a new line of study that allows us to deepen the relationship between NANOG and *CXCR4* in glioblastoma cells. In this way, it would be very interesting to separate NANOG-positive subpopulations from each glioblastoma cell line and then compare them side-by-side to their corresponding NANOG-low/negative counterparts, for the analysis of *CXCR4* expression levels and migratory abilities.

In conclusion, our results suggest that the role of NANOG in cancer stem cell migration may be due to the direct regulation of the *CXCR4* gene. Thus, we provide a novel mechanism connecting two different concepts in cancer: pluripotency in cancer stem cells with invasiveness, tumor progression and drug resistance. As *NANOG* is not expressed in most adult tissues, our findings identify NANOG as a potential therapeutic target in the treatment of *NANOG*-expressing metastatic cancers.

## 4. Materials and Methods 

### 4.1. Fish Embryos 

Adult medaka (*Oryzias latipes*) CAB strain animals were kept in recirculating water aquaria at 28 °C on a 14 h light/10 h dark daily cycle. Embryos were collected by natural spawning in 1× Yamamoto [65] and staged as described [66]. Embryos were raised at 25 °C.

### 4.2. ChIP Assay 

Five hundred embryos at stage 16 were homogenized in 1% paraformaldehyde (PFA; Sigma-Aldrich, St. Louis, MO, USA)/phosphate buffer saline (PBS; Sigma-Aldrich, St. Louis, MO, USA) solution containing protease inhibitors using a mortar and pestle, fixed in this solution for 8 min, and washed with cold PBS containing protease inhibitors. The solution was filtered and centrifuged at 470 g for 10 min at 4 °C, and cells were stored at −80 °C. Thereafter, ChIP was performed as previously described [40]. Samples were incubated with magnetic beads conjugated to medaka specific anti-Ol-Nanog [42], anti-Ol-Oct4 [67] or anti-Ol-Ptc1 antibodies [40]. After magnetic capture and DNA release, the DNA was precipitated and resuspended in 50 μL of water for PCR analysis. The DNA fragments were then subjected to analysis by RT- PCR (Bio-Rad, CA, USA) in triplicate using 1 µL of DNA solution per reaction and primer pairs indicated in Supplementary Table S3. The annealing temperature used for primer pairs was 57 °C and 32 cycles were used.

### 4.3. mRNA and Morpholino Injection

The h-Nanog cDNA was amplified using the following primers (Sigma-Aldrich, St. Louis, MO, USA): h-NANOG F, ATGAGTGTGGATCCAGCTTG; h-NANOG R, TCACACGTCTTCAGGTTG and was cloned in pCS2 to obtain pCS2-h-NANOG.

pCS2-Ol-Nanog [42] and pCS2-h-NANOG were used for mRNA synthesis with the SP6 Ambion mMessage mMachine Kit (Invitrogen; Waltham, MA, USA). The MO (Gene Tools LLC, Philomath, OR, USA) sequences are: Nanog MO 5′-TGACCTGAGTTTTCCACTCCGCCAT-3, and control MO (MO-C) 5-CCTCTTACCTCAGTTACAATTTATA-3′ [40,42]. MOs at a concentration of 0.5 mM and synthesized mRNAs at a concentration of 200 ng/µL were injected in one cell of medaka embryos at st. 1 using a pressure Narishige IM300 microinjector (Narishige International Limited, London, UK).

### 4.4. In Silico Searches for Nanog Binding Sequences

Medaka −3265 bp and human −8530 bp upstream regions were retrieved from ENSEMBLE (https://www.ensembl.org/index.html). Potential Nanog DNA binding sequence were searched with published binding consensus motif [43] using the Regulatory Sequence Analysis Tools (RSA) pattern Matching DNA-pattern software (http://rsat.ulb.ac.be/rsat/). In addition, the human CXCR4 upstream sequence was also analysed with Genomatix Matinspector software (https://www.genomatix.de/) [68].

### 4.5. Cell Lines

293T cells (Sigma-Aldrich; St. Louis, MO, USA) were maintained in Dulbecco´s modified Eagle´s medium + GlutaMAX (Ref 31966, Gibco; Dublin, Ireland) supplemented with 10% heat inactivated fetal bovine serum (FBS, Life Technologies; Carlsbad, CA, USA) and penicillin/streptomycin (Ref. 15140122, Life Technologies; Carlsbad, CA, USA). Human malignant glioma cells U-87, U-251 and U-373 were also supplemented with 1xMEM non-essential amino acids (Ref 11140, Gibco; Dublin, Ireland).

### 4.6. Reporter Assay

Medaka expression constructs were obtained after cloning the oligo sequences containing one of the Ol-Nanog binding sites of *Ol-Cxcr4* or the corresponding mutated sequence (see Supplementary Table S4 for oligo sequences and Supplementary Figure S1 for sequence positions in medaka genome) in the kpnI site of the pGL3 luciferase reporter vector (Promega; Madison, WI, USA). Every oligo sequence had 4 repetitions of the corresponding Nanog binding site. To easily identify constructs, the NotI site was included before the oligo sequences. Final constructs were verified by sequencing with RVprimer3 and GLprimer2.

Human expression constructs were obtained after cloning the oligo sequence containing the predicted h-NANOG binding site of *h-CXCR4* (position chr2: 136, 876, 177–136, 876, 202) or the mutated sequence 1 (see Supplementary Table S1 for oligo sequences) in the kpnI site of the pGL3 luciferase reporter vector (Promega; Madison, WI, USA). Mutated sequence 2 and double mutated 1 + 2 constructs were obtained using the QuikChange II XL Site-Directed Mutagenesis Kit (Stratagene; Bellingham, WA, USA) following the manufacturer instructions (see Supplementary Table S2 for oligo sequences). To easily identify constructs, the NotI site was included before the oligo sequences. Final constructs were verified by sequencing with RVprimer3 and GLprimer2.

The transcription activity of the human and medaka CXCR4 promoter in 293T cells was determined by luciferase reporter plasmid assay. Cells were plated at a density of 50,000 cells/well one day before transfection. Then, 24 h later, cells were transiently transfected with pGL3 promoter reporters (0.2 µg) and effector plasmids (medaka or human Nanog expression construct, 0.2 µg) using FuGENE HD (Ref. E2311, Promega; Madison, WI, USA) according to the manufacturer’s instructions. pBS empty vector was included to normalize the amount of total DNA for each transfection. pCMV-Renilla (20 ng/transfection) was included in each transfection as an internal reference. Then, 48 h after transfection, cells were washed with PBS and lysed in the lysis buffer provided with the luciferase kit. Transcription activity was measured using the dual luciferase reporter assay system (E1910, Promega; Madison, WI, USA) in a luminometer for plates (Berthold Technologies; Oak Ridge, TN, USA). Each transfection was done in triplicate.

### 4.7. Production and Usage of shRNA Virus

Non-silencing-GIPZ lentiviral shRNAmir (Open Biosystems, ref: RHS4346; Huntsville, AL, USA) and GIPZ-shNanog (Open Biosystems, ref: RHS4430-98842347; Huntsville, AL, USA) were cotransfected with the 2nd generation packaging vectors: psPAX2 and pMD2.G (courtesy of D. Trono´s lab) into the 293T producing cells. Cells were refeed with new media 12 h later and the viral supernatant was collected 2 days after transfection. The supernatant was centrifuged at 3000 rpm during 10 min and filtered through 0.45 μm syringe filters. Aliquots of the diluted virus were kept frozen at −80 °C and used 1:2.

### 4.8. Transfection and Transduction Experiments

To determine NANOG expression level in U-87, U-251 and U-373 cell lines, each cell type was plated in 24 well plates (50,000 cell/well) for 48 h. After the incubation period, total RNA from cells was extracted. This experiment was done in triplicate.

For transfection experiments, human malignant glioma cells U-87 were plated in 24 well plates (50,000 cell/well) 24 h prior to transfection. Transfection mixes were prepared with FuGENE HD (Ref. E2311, Promega; Madison, WI, USA) and 0.45 µg of pCS2-h-NANOG, or 0.45 µg of pCS2 control vector, or without any DNA. Total RNA from cells was extracted 48 h post transfection. This experiment was done in triplicate.

For transduction experiments, human malignant glioblastoma cells U-251 and U-373 were seeded in 24-well plates (50,000 cells/well) overnight and transduced with shRNA-hNANOG virus, or shRNA-control virus, or without any virus, in the presence of Polybrene (5 µg/mL; Hexadimethrine Bromide; Sigma, St. Louis, MO). Cells were collected 1 day post transduction for RNA extraction. This experiment was done in triplicate.

Total RNA extraction from cells was performed using Trizol reagent (Ref. 15596-026, Invitrogen, Carlsbad, CA, USA) and treated with DNA-free kit (Applied BioSystems, Foster City, CA, USA). cDNA was synthesized from 1 μg of total RNA using random primer hexamers (Ref. 11034731001, Roche Diagnostics; Basel, Switzerland) and Superscript III reverse transcriptase (Ref. 18080044, Life Technologies).

### 4.9. qPCR Analysis for Cell Samples

For quantitative reverse transcriptase reaction (qRT-PCR), we used 1.7 ng of cDNA in 1xSYBR Green Master Mix (Ref. 4367659, Applied Biosystems, CA, USA) and 0.25 mM each of forward and reverse primers. Primer pairs were as follows: hNANOG F 5′ AGAAGGCCTCAGCACCTAC 3′, hNANOG R 5′ GGCCTGATTGTTCCAGGA 3′, hCXCR4 F 5′ TGGCCTTATCCTGCCTGGTAT 3′, hCXCR4 R 5′ GGAGTCGATGCTGATCCCAAT 3′, hCXCR7 F 5′ TGGCGGTGCTGCTGGACA 3′, hCXCR7 R 5′ GCACCAGCGACAGGCACT 3′, hACTIN F 5′CACAGAGCCTCGCCTTTG 3′, hACTIN R 5′CCATGCCCACCATCACGC 3′. The PCR amplification and fluorescence detection were performed in a real-time PCR thermal cycler (Viia7 Real-Time PCR system, Applied Biosystems). The PCR conditions were 40 cycles of denaturation at 95 °C for 15 s and annealing at 60 °C for 30 s. The melting curve was constructed by plotting fluorescence data against temperature (55−95 °C). Control samples without cDNA were included in all assays to confirm the absence of nonspecific amplification products. For each sample, the threshold cycle (Ct) for the internal control (*hACTIN*) amplification was subtracted from the threshold cycle of the corresponding transcription factor amplification (Ct, transcription factor) to yield ∆Ct. The Pfaffl method was used to calculate the ratio of relative gene expression related to *hACTIN* (internal control) and is represented in the bar graphs. Primer pair efficiencies were calculated for each primer pair by performing dilution curves (*hNANOG* 1.9, *hCXCR4* 1.86, *hCXCR7* 1.98 and *hACTIN* 2). qRT-PCR reactions were performed in triplicate for each experiment. Bar graphs represent the results from three independent experiments.

### 4.10. cDNA Preparation and qRT-PCR from Human Samples

Total RNA from human tissues was extracted using the RNeasy Kit (Qiagen; Hilden, Germany), and it was digested with RNase free DNase I (Qiagen; Hilden, Germany) according to the manufacturer’s instructions. Total RNA (1 μg) was reverse transcribed with SuperScript II Reverse Transcriptase (Invitrogen; Waltham, MA, USA).

qRT-PCR was performed using the Light Cycler 1.5 (Roche; Basel, Switzerland) with the SYBR Premix Ex Taq (Takara; Saint-Germain-en-Laye, France) and using HPRT (F 5′ TGACACTGGCAAAACAATGCA; R 5′ GGTCCTTTTCACCAGCAAGCT) as an internal control of expression. The primers used for NANOG were hNANOG F 5′ AGAAGGCCTCAGCACCTAC 3′, hNANOG R 5′ GGCCTGATTGTTCCAGGA 3′. Reactions were performed in LightCycler^®^ Capillaries in a final volume of 10 μL containing: SYBR Premix Ex Taq II (5 μL) (Takara; Saint-Germain-en-Laye, France), 10 μM forward and reverse primers (0.2 μL), 2 μL of cDNA template (ten-fold diluted) and nuclease-free water (2.6 μL). Cycling conditions included an initial denaturation step of 10 min at 95 °C, followed by 45 cycles of 10 sec at 95 °C, 10 sec at the primer hybridization temperature (55 °C for HPRT and 59 °C for NANOG) and 10 sec at 72 °C. The cDNA from normal tissue, adjacent to one of the tumors, was used to calibrate all RT-PCRs. Gene expression was quantified by the delta-delta Ct method.

### 4.11. Transwell Assay

U-87 cells were plated in 6 mm plates in a density of 7 × 10^5^ cell/well 24 h prior to transfection. Transfection mixes were prepared with FuGENE HD (Ref. E2311, Promega; Madison, WI, USA) and 0.45 µg of pCS2-h-NANOG, or 0.45 µg of pCS2 empty vector. The cells were harvested and resuspended in serum-free GlutaMAX (supplemented with 1xMEM non-essential amino acids; Gibco; Dublin, Ireland) 48 h post transfection. Cells from each transfection were added to different upper chambers of the HTS-24 Multiwell Insert System plate (10,000 cells/chamber; Ref: 351185; BD Falcon; Franklin Lakes, NJ, USA) and 750 μL of serum-free GlutaMAX to lower chambers. Then, 24 h later, the media was removed and the following culture conditions were added to the chambers in triplicate: for cells transfected with the empty vector, one condition was 0.5% FBS-GlutaMAX in upper and lower chambers; the second condition was 0.5% FBS-GlutaMAX in upper chambers and 10% FBS-GlutaMAX in lower chambers; the third condition was 0.5% FBS-GlutaMAX plus 10 µM AMD3100 (Ref A5602; Sigma-Aldrich; St. Louis, MO, USA) in upper chambers and 0.5% FBS-GlutaMAX plus 0.05 µg/mL SDFα (Ref 300-28A; Bionova, Madrid, Spain) in lower chambers; and the last condition was 0.5% FBS-GlutaMAX in upper chambers and 0.5% FBS-GlutaMAX plus 0.05 µg/mL SDFα in lower chambers. For cells transfected with pCS2-h-NANOG vector, one condition was 0.5% FBS-GlutaMAX in upper and lower chambers, the second condition was 0.5% FBS-GlutaMAX in upper chambers and 0.5% FBS-GlutaMAX plus 0.05 µg/mL SDFα in lower chambers, the third condition was 0.5% FBS-GlutaMAX plus 10 µM AMD3100 in upper chambers and 0.5% FBS-GlutaMAX in lower chambers, and the last condition was 0.5% FBS-GlutaMAX plus 10 µM AMD3100 in upper chambers and 0.5% FBS-GlutaMAX plus 0.05 µg/mL SDFα in lower chambers. Plates were incubated at 37 °C for 4 h. After incubation, supernatants were discarded, membranes fixed with 4% formaldehyde for 20 min, and cells from upper chambers were removed with cotton buds. Cells that migrated throughout the membrane were stained with DAPI. Finally, four random fields per transwell were counted. This experiment was done in triplicate.

### 4.12. Statistical Analysis

Statistical analysis to determine significant changes were performed on GraphPad Prism (Version 4.00, 1992–2003 GraphPad Software Inc.; San Diego, CA, USA) using one-way ANOVA plus Tukey post hoc test. For all data, a level of 5% or less (*p* < 0.05) was taken as statistically significant.

## Supplementary Materials

The following are available online at https://www.mdpi.com/article/10.3390/ijms221910620/s1.
