# Supplementary material for: The Embryonic Key Pluripotent Factor NANOG Mediates Glioblastoma Cell Migration via the SDF1/CXCR4 Pathway
Source: Int J Mol Sci. 2021 Sep 30;22(19):10620. doi: 10.3390/ijms221910620 (PMC8508935; doi:10.3390/ijms221910620)
Supplement: Supplementary file 1 [file ijms-22-10620-s001.zip › Suppl. Fig. 1.pdf]

**Supplementary Figure S1.**

Putative Nanog binding sequences in the *Ol-Cxcr4b* gene upstream region were Ol-Nanog binding was previously demonstrated by ChIP assay (Sánchez-Sánchez et al., 2010). In blue, bold and underlined putative Nanog binding sequences 1-3. (G/A) (G/C) ATTA (G/AT) (GC). Underlined only pairs of primers that amplified bands in the ChIP experiment performed in Sánchez-Sánchez et al., 2010.

ultracontig:MEDAKA1: ENSEMBLE Genomic location:
ultracontig257 780993 to 784260 (+)

GCAGCCGAAGGTCGGCCTTGCCGAGTTCTGCCGCAGGTTTCTTCTTTGTCTTTTATTTAG
TGGAAGCCGTTCTCCCCACAGTCGCCAGCTGCTGTTTTCCAGACAAAGAAGTGCAATGAA
ACCCTGCAGGACTATTTTAAAAATAAAATCAAGACATTTTAGCCTCATCAGAAAGATCTG
GCCTGAGTTGGATCAAAATAATCCTAATTATTGAATTATTAAACACATGATGTGTTTAAT
GCAGTGAATGATTCGATTTTAGGCCACAAAAGTGACCTTAGATTATCAACACCCACCTTT
GAAAAACACACAAAGTCAGCGTGTCTAATCAAAACTAAGTGCCTGCACCAAGTTTTGATT
CTGAAAAAGAAAAAATTGAACTTTGAGTCTGACTGTCACACGGAATAATGATCTCATCTT
TTGGTTGCGCTTTGGAATTGCTCATTCTTGTC***TTAATCTC***AATGCCGCTGCCTCTTTTCA
ATGGCCATTCTTCACTCAAGAGGCCCTTTTCGCTGCCGTTCACACTCCTGGCGATTTGTA
TTTGAAGTGGGTTGCTGATCTTTTGTATTCTGGACTGATAGCGATCTGAGTCCTTGGGCT
CACGCAGGAGCTCCAGGTATGCAACCTCCAGGAGTTCATGTGGAAACTGAAGTCTGCTCT
GTTGAAACTCAGTGAAATGATGAGCTAAAACCCACAAAACATCAAAAAGCTAATTGCGTA
AGTGAGCTGATTGCAGAGAGCTGATTATGTCTGCATTTATTCAGATTTATAAAGCAGAAC
ACAAATACTCTTGAGCTTGAGAAAACGTGAACACGTTGTGAAACGTTTATTGCAATAAAC
ATGCACTTTTCTCTTTAAAAACTCTGACTTTTAAGTATCTGGCCCTTTTAATCATTGCAA
CCTCCCAACTCCAACAGATTTATTTGAATTTAAAAGGAAATCATGACAAAACTTGAAATG
GATAGAAACATTCACATGCTCAGAAGGCGATGTGCCAGAGAACAACCTTAAAAACAGAAA
TTTTCTCTTCCTCAGGACATAAAATTAAACAAAATGCTGCGCATAATT***CAGATTAA***AAGT
GACGGCTTTGCTCACAGGCAGGTACAGCAGCAACTGTCTGTGTCTGAATGCCATTGATAA
GAGCCTCATCCCCATACAGTGGCCTTTCTCTGAGCTGCCACCTCCTTGGGTTTCCTCTTC
TTGGACTGCAGGGGTTCCTGAACGACTCCTCCAACTGCTTTGATAAGCAGCACGTGATTT
AGAAGCAGCGGTGTGCGGTTGCTGCCTGTCATTTGTTGCTCGTTATTTTATGGCAAACCA
ACAAATGTTGTGGTTTTTGTAATGAAGCAACAGTCTCTGAGCACCAGTGTTTGTGCCTCA
CGTCTTCTCAGGAAAGGGAAAATGTTCTTCTGGGGGGGGGATTTCAGACTTCAGCACCTG
GCTGAACAGAAAAGGAACTTCTATGATGTCCTAGAGAGCATTCGGAAGAAAACCCACAAC
ACAAATGGACTTTCCTGATTCAACCAATCATTTCACAAACGTTCCCAGTTCAATGAACAC
CCATAATGCACCTGCAATACTTTCACTGCAAAAACAGGCAGCCTACAATAGGAAAAGAAC
TCTAATCTTAAGAAGATCACTGGAAAGTAGTGAGATGATCTGTCCGTGCTGAAGGTCATT
TTTACAGAACAAGCAATCTCATAATAAGAAATCAAAATAGGTTCCATGCAAGCACTCAAT
ACGTACGCTAAAGAATCTGTTAGAGTTCTTCAAACTGAATATTCTGCAGCCCAAAAATAA
GTATTTTTCCACTTGAAAGCAAACGGTTTGTAATTTATTTGTGGTGTTGTTGAGTTTTTA
TGGACCTAATAGGATTAAAGTCTCAAAATACTAGTGTATGAGTGGAAATTACTCATTTTT
AGAAATAAATGTGGACAAAATGAGAATTTAAGATCTATTGAAAAAATCTAAAATCACCAC
TTATTTTTAGAACGTAATTCTACATTTTTCCCCCTAAAATAATGACAAGGAATGCCAGTC
TAATTCATCAACATTTGTTGAACTCTGAAATAACATCCTTAAATTCTTTGTTTAATATCT
GAAGCAAGAATTCCAGTGTAATTCCAGTTTTTTTGAGAATTATATCAAGGTTTTCTATTG
CCAAGAGTTTGGAGCCAAAGGATTTGGACTAATTTCCTCTAGTTCTTCATGATTTAGAGA
GAAGCCTGGAAAGGTGTGTAGCACAGAGAAGTTAAACAACTTCAAACCTGCTGAAACATC
TTGGTGGTCCGGTTTGAAGTCTTTGCCCGTATCACATAGTTTTATGAAGCGAGGGTGTGC
TTATCGATGAATCCCATCTAAAAGGCAGTTGCTGCTGCTAAATCTTGAACCAAACTTTCA
CACATTGACTCGGCTGAGGAAGGAAATCGCAAAAAGGCAATTTGATCTTTTTTTTGTGTC
AAAGCTTCGTGTCTGGAAATGGTGATCTCTCCTTGTTCCAACTGTGGATGAACAGATCCC
AATGATCTATCATATATCAAAGGCATTGAGGGGCGTTTGCATGCCACATGGAGGATATTG
AGTTTTGAAATTAGAGTCGGGTGCAGAGTTGGCCCCCTGGTCACAGACAGAGAGCTGCCT
GACATCAACCC***CACATTAA***AAGGTTCATTAAACACTTTGGAGCGCAGACATTGTAGTTGA
GAAGTGACAGAAGGTGGGGAGAGAAAAAAAGGTGAGGGCATGTCGGAACGCCCCCTGACA
GAAGAGGAGGTGAGGAAGGTGAGGAAGGTGAAGCTGCCAGGAAGGTGAGGCTCCCAGAAA

GGTGAGGCTGCCAGGAAGCTGTCCTTCCGTCCGCCTGCAAGAGCCAGAGCTCCTGAAATC
CTGTGAATGCCCCAAAACCTTCCAAACAGAGCATCTTCAAAGCTATTGCCTTTTGTTCTG
ATCTAAATTCCTGCTAAAATGAAAAAAACTCTAAAGCTGCAGAGAAGAAACTTCTTTGGA
GGCATAAAAACAAAACTTCACGTTTTTTTCCTCAGCGCATTTGCTGGTTTTGGACGCCAG
GAGATGGCAGACGCCTCCAGCTCTGACTGACAGGACAGAGCGGCACAAAGAGACGCCCCC
TCGCACGCGCCTTTTATACTGCAGCCGTTTTTGGAGCGCGGTGGGCGGGGCCAGGGGCCC
CCGCGCGGGGACCGTACTTATTCAGACAAACAACCTGAACGGCTCATTCTGCACTCATCC
TGACGGAGGGACACCGGCAGCGACCATG
