# Supplementary material for: The Embryonic Key Pluripotent Factor NANOG Mediates Glioblastoma Cell Migration via the SDF1/CXCR4 Pathway
Source: Int J Mol Sci. 2021 Sep 30;22(19):10620. doi: 10.3390/ijms221910620 (PMC8508935; doi:10.3390/ijms221910620)
Supplement: Supplementary file 1 [file ijms-22-10620-s001.zip › Suppl. Fig. 2.pdf]

Supplementary Figure S2

Putative NANOG binding sequences in the *Hs-CXCR4* gene. A) Putative NANOG binding sequences: Highlighted in green Genomatix Matinspector and in bold blue according to the motif (G/C) N (G/T) AA (G/T) (G/C). B) Comparison of the human CXCR4 putative NANOG binding DNA sequence with those reported in mouse *Rex-1* and human *CDK6* and *CDC25A* genes. Highlighted in green equal in all sequences and highlighted in yellow equal to the CXCR4 sequence.

A)

>CxCR4 upstream sequence. ENSEMBLE Genomic location: Chromosome 2 136875621 to 136877022 (+)

GGTCCGTGTCGCGACGCACGCGCCTCGGTCCCAGCTATCTCCGCAGCGGCCACCCCGCCTGCGGACGCAGTTTCTCGGCCCCGCCC
CACACTCGCTCCCCCGCCCCACCCAGTCTCCGCGCCGGAGGGAAGTGGCGCGAGGGGGAAAGCACTGTCTGCGCGCCCACTGCAAA
CCTCAGCCAGTCTGAGATCGCTTTAAACGTCTGACCCCCACCCCCACTCCGCCCCGCCCAGTTCTTCAACCTAATTTCTGATTCGT
GCCAAAGCTTGTCCTCTGCTCAAAATCGTGGAAGACGCCGAGTATGGGGACCGAAGACCTGGGTTCAAGCCCGGCTTGGAATCCCT
GCCCATCCCTGGCATTTCATCTCTCCGGGCTTATTTGCTGGTTTCTCCGAATGCGGGCCTTGTCTGGTTCACGCTGGATCCCCAAC
GCCTAGAACAGTGCGTGGCACGCAGTTCGTCCTTCTATAAATATCGGACTAAATGCATCTCTGTGATGGTAATACCCACACGGTGT
TGTGAGAATGAATGAGTGATTCTGTGCAAGTTCCTAGTGATCTGTTACAAAAAGTACTGGTCGCTAAATTACTCTTATAATAAAGC
ATACTTTTAGGATAATAAAGCACTATTCGCGAATTGGTTACCGCTATTATGAAATTACTGAGCAATACATATCTACATCTGATCAG
TCTCCAGAATTATGCCAAATCCTACCTTCTTCTGAAAGTATCTCCTAATTATCTGCACCTGACCCTAGTGATGCTGTGAATGTGCA
AGTATAGCTACATCCTCCGAAGGAAGGATCTTTACTCCTTTTACCTC**CTGAATG**GGCTGCGTCTG**CTGAAAG**CGCG**GGGAATG**GCG
TTGGAAGCTTGGCCCTACTTCCAGCATTGCCGCCTACTGGTTGGGTTACTCCAGCAAGTCACTCCCCTTCCCTGGGCCTCAGTGTC
TCTACTGTAGCATTCCCAGGTCTGGAATTCCATCCACTTTAGCAAGGATGGACGCGCCACAGAGAGACGCGTTCCTAGCCCGCGCT
TCCCACCTGTCTTCAGGCGCATCCCGCTTCCCTCAAACTTAGGAAATGCCTCTGGGAGGTCCTGTCCGGCTCCGGACTCACTACCG
ACCACCCGCAAACAGCAGGGTCCCCTGGGCTTCCCAAGCCGCGCACCTCTCCGCCCCGCCCCTGCGCCCTCCTTCCTCGCGTCT
GCCCCTCTCCCCCACCCCGCCTTCTCCCTCCCCGCCCCAGCGGCGCATGCGCCGCGCTCGGAGCGTGTTTTTATAA
AAGTCCGGCCGCGGCCAGAAACTTCAGTTTGTTGGCTGCGGCAGCAGGTAGCAAAGTGACGCCGAGGGCCTGAGTG
CTCCAGTAGCCACCGCATCTGGAGAACCAGCGGTTACC**ATGGAGGGGA**

B)

h-CXCR4	CCTG**AA**TGGGCTGCGTCTGCTG**AA**AG
m-Rex-1	AGTTATGCAAATGCCTCTTCAGAATC
h-CDK6	CTGCTTCTTTATGGATCCACTTAATA
h-CDC25A	AGCCAGGCCGGCCTTTCGCGGTAATA
