# Supplementary material for: The Embryonic Key Pluripotent Factor NANOG Mediates Glioblastoma Cell Migration via the SDF1/CXCR4 Pathway
Source: Int J Mol Sci. 2021 Sep 30;22(19):10620. doi: 10.3390/ijms221910620 (PMC8508935; doi:10.3390/ijms221910620)
Supplement: Supplementary file 1 [file ijms-22-10620-s001.zip › Supplementary Table 1.pdf]

Supplementary Table S1

	**Forward primer**	**Reverse primer**	**Product length bp**	**Fragment position**
***Ol-Nanog* regulatory region**	CTGCATGGTTTGCGCTTC	TGCATAAATGGTGTGCATGG	168	chr20:10,397,140-10,398,200
***Ol-Nanog* no regulatory region**	TCTGGCTCCAACATGGCG	ATGAGGCGGCAATCAACG	236	chr20:10,400,500-10,401,300
***Ol-Oct4* regulatory region**	TTATTGTTCTGATGTGAAAAGG	AAACCCTTCTGCTTTCAG	245	chr12:15,547,100-15,548,500
***Ol-Oct4* no regulatory region**	GGAATCAGGGCAGAATTC	CTGTCAACACTGATCTGGG	260	chr12:15,545,240-15,546,530
***Ol-Sall1* regulatory region**	GTGTGTGATGAAAACGTG	GCATTAAGATTGTTGTAATTCG	254	chr3:29,316,100-29,316,950
***Ol-Sall1* no regulatory region**	CATATGACTTCTGCTGTGTCC	AGCAAAAAGATGTGAAAAACCC	147	chr3:29,313,900-29,314,450
***Ol-Sall4* regulatory region**	TGCCATGAAGGACGAAAG	TTGCGATTTATCACGAGC	182	chr7:25,177,400-25,177,850
***Ol-Sall4* no regulatory region**	ACCAAGACTCGTCAACTGG	TCACCAAACTGGGAATCAC	267	chr7:25,174,741-25,175,642
***Ol-p53* regulatory region**	CTTTGGTAACATAACTCTGG	CTGCATTTAGTTGGAGTCAC	170	chr: 18. 14431941 -14432606
***Ol-p53* no regulatory region**	GGGTATGTGAGTGAAAAGG	TGTATATTGAAGCCGTGC	236	chr: 18. 14446500-14447000

Oligo sequences used for ChIP amplification.
