# Supplementary material for: The Embryonic Key Pluripotent Factor NANOG Mediates Glioblastoma Cell Migration via the SDF1/CXCR4 Pathway
Source: Int J Mol Sci. 2021 Sep 30;22(19):10620. doi: 10.3390/ijms221910620 (PMC8508935; doi:10.3390/ijms221910620)
Supplement: Supplementary file 1 [file ijms-22-10620-s001.zip › Supplementary Table 2.pdf]

Supplementary Table S2

Medaka oligo sequences	KpnI/NotI/4xNanog binding site/TATA/KpnI
1.4xNanog+<br>1.4xNanog-<br><br>1.4xNanog.m+<br>1.4xNanog.m-	5′ C GCGGCCGC TTAATCTC TTAATCTC TTAATCTC TTAATCTC TATAAA GGTAC 3′<br>5′ C TTTATA GAGATTAA GAGATTAA GAGATTAA GAGATTAA GCGGCCGC GGTAC 3′<br><br>5′ C GCGGCCGC TTCCTCTC TTCCTCTC TTCCTCTC TTCCTCTC TATAAA GGTAC 3′<br>5′ C TTTATA GAGAGGAA GAGAGGAA GAGAGGAA GAGAGGAA GCGGCCGC GGTAC 3′
2.4xNanog+<br>2.4xNanog-<br><br>2.4xNanog.m+<br>2.4xNanog.m-	5′ C GCGGCCGC CAGATTAA CAGATTAA CAGATTAA CAGATTAA TATAAA GGTAC 3´<br>5′ C TTTATA TTAATCTG TTAATCTG TTAATCTG TTAATCTG GCGGCCGC GGTAC 3′<br><br>5′ C GCGGCCGC CAGACCAA CAGACCAA CAGACCAA CAGACCAA TATAAA GGTAC 3′<br>5′ C TTTATA TTGGTCTG TTGGTCTG TTGGTCTG TTGGTCTG GCGGCCGC GGTAC 3′
3.4xNanog+<br>3.4xNanog-<br><br>3.4xNanog.m+<br>3.4xNanog.m-	5′ C GCGGCCGC CACATTAA CACATTAA CACATTAA CACATTAA TATAAA GGTAC 3′<br>5′ C TTTATA TTAATGTG TTAATGTG TTAATGTG TTAATGTG GCGGCCGC GGTAC 3′<br><br>5′ C GCGGCCGC CACACCAA CACACCAA CACACCAA CACACCAA TATAAA GGTAC 3′<br>5′ C TTTATA TTGGTGTG TTGGTGTG TTGGTGTG TTGGTGTG GCGGCCGC GGTAC 3′
