# Supplementary material for: The Embryonic Key Pluripotent Factor NANOG Mediates Glioblastoma Cell Migration via the SDF1/CXCR4 Pathway
Source: Int J Mol Sci. 2021 Sep 30;22(19):10620. doi: 10.3390/ijms221910620 (PMC8508935; doi:10.3390/ijms221910620)
Supplement: Supplementary file 1 [file ijms-22-10620-s001.zip › Supplementary Table 3.pdf]

Supplementary Table S3

Human oligo sequences	KpnI/NotI/Nanog binding site/TATA/KpnI
HsCxcr4 promotor+	5´ CGCGGCCGCCCTGAATGGGCTGCGTCTGCTGAAAGTATAAAGGTAC 3´
HsCxcr4 promotor-	5´ CTTTATACTTTCAGCAGACGCAGCCCATTCAGGGCGGCCGCGGTAC 3´
**Human oligo sequences**	KpnI/NotI/Sequence mut 1/TATA/KpnI
HsCxcr4 mut 1+	5´ CGCGGCCGCCCTGTCCAGGCTGCGTCTGCTGAAAGTATAAAGGTAC 3´
HsCxcr4 mut1 -	5´ CTTTATACTTTCAGCAGACGCAGCCTGGACAGGGCGGCCGCGGTAC 3´
