# Supplementary material for: The Embryonic Key Pluripotent Factor NANOG Mediates Glioblastoma Cell Migration via the SDF1/CXCR4 Pathway
Source: Int J Mol Sci. 2021 Sep 30;22(19):10620. doi: 10.3390/ijms221910620 (PMC8508935; doi:10.3390/ijms221910620)
Supplement: Supplementary file 1 [file ijms-22-10620-s001.zip › Supplementary Table 4.pdf]

Supplementary Table S4

HsCxcr4 mut 2	Primer Sequence (5' to 3'). Mut 2
HsCxcr4 mut 2 +	5'-cctgaatgggctgcgtctgctcggagcgcggggaa-3'
HsCxcr4 mut 2 -	5'-ttccccgcgctccgagcagacgcagcccattcagg-3'

HsCxcr4 mut 1+2	Primer Sequence (5' to 3'). Mut1 + Mut2
HsCxcr4 mut 1+2 +	5'-cctgtccaggctgcgtctgctcggagcgcggggaa-3'
HsCxcr4 mut 1+2 -	5'-ttccccgcgctccgagcagacgcagcctggacagg-3'
